# Three New Steroidal Glycosides from the Roots of *Cynanchum stauntonii*

**DOI:** 10.1155/2013/816145

**Published:** 2012-12-26

**Authors:** Jin-Qian Yu, Zhi-Hui Zhang, An-Jun Deng, Hai-Lin Qin

**Affiliations:** State Key Laboratory of Bioactive Substance and Function of Natural Medicines, Institute of Materia Medica, Chinese Academy of Medical Sciences and Peking Union Medical College, Beijing 100050, China

## Abstract

Three new steroidal glycosides, named as stauntosides L, M, and N (**1–3**), along with one known C_21_ steroidal glycoside, anhydrohirundigenin monothevetoside (**4**), were isolated from the 95% ethanol extract of the roots of *Cynanchum stauntonii*. The structures of these new compounds were elucidated on the basis of extensive spectroscopic analyses, mainly 1D and 2D NMR, HRESI-MS, and chemical methods.

## 1. Introduction

**Figure 1:**
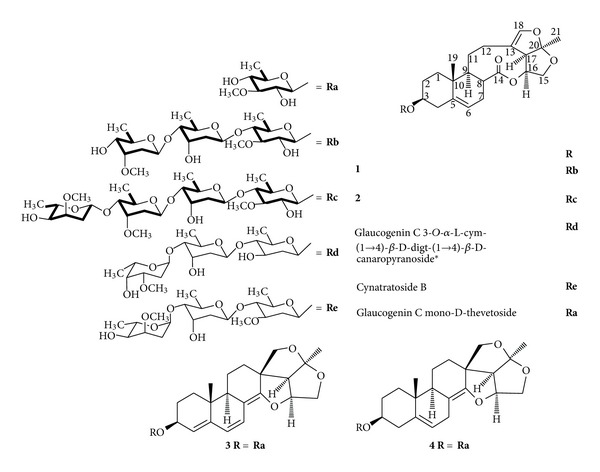
The structures of compounds **1–4 **and the reference compounds, and the key ^1^H, ^1^H-COSY correlations in oligosaccharide moieties. (—: ^1^H, ^1^H-COSY). *cym: cymaropyranosyl; digt: digitoxopyranosyl.

*Cynanchum stauntonii *(Decne.) Schltr. ex Levl. is a perennial medicinal herb from the family of Asclepiadaceae, which is widely distributed in south-central region of China. The dried-up roots of *C. stauntonii*, along with that of another species of the same genus, *C. glaucescens* (Decne.) Hand.-Mazz., has been used as antitussives and expectorants to treat diseases in the history of China [1]. Both of which are given the name of “Bai-qian” in traditional Chinese medicine (TCM) [2]. The main chemical constituents isolated from *Cynanchum* species are steroids, especially the steroidal saponins with aglycones assignable to either the normal four-ring C_21_ steroid skeleton or the aberrant 13,14 : 14,15-disecopregnane-type skeleton or the equally abnormal 14,15-secopregnane-type skeleton, respectively [3, 4]. It is known that C_21_ steroids and their glycosides are of considerable bioactivities, such as hypolipidemic and antitumor activities. However, chemical investigation into the title plant is very rare up to now with, to the best of our knowledge, only three papers have reported several steroids, including four ones by our group eight years ago [1]. The ongoing investigations in our group intend to enrich the information about the chemical constituents and their bioactivities of this plant which has led to the isolation and elucidation of some known and new steroidal glycosides [5]. In this paper, we describe three new steroidal glycosides (**1–3**) and one known analogue, anhydrohirundigenin monothevetoside (**4**) (Figure 1), from the roots of *C. stauntonii*. The isolated new steroidal glycosides contained steroid aglycones with either the 13,14 : 14,15-disecopregnane-type skeleton or the 14,15-secopregnane-type skeleton and were given the trivial names stauntosides L-N, respectively.

## 2. Materials and Methods

### 2.1. General Methods

Optical rotations were measured on a Perkin-Elmer 241 digital polarimeter at 20°C. IR spectra were recorded on a Nicolet 5700 spectrometer. 1D and 2D NMR spectra were taken on a Varian INOVA-500 spectrometer or a Varian NMR System-600 NMR spectrometer with tetramethylsilane as internal standard. ESIMS and HRESIMS were obtained using an Agilent 1100 series LC/MSD Trap SL mass spectrometer. Preparative HPLC was performed on a Shimadzu LC-6AD system equipped with a SPD-10A detector, and a reversed-phase C18 column (YMC-Pack ODS-A U 20 × 250 mm, 10 *μ*m) was employed. Column chromatography (CC) was undertaken over silica gel (200–300 mesh). TLC was carried out with glass plate precoated silica gel G. Spots were visualized under UV light and by spraying with 10% H_2_SO_4_ in 95% EtOH followed by heating. GC was conducted on an Agilent 7890A instrument. Reference compounds, glaucogenin C 3-*O*-*α*-L-cymaropyranosyl-(1→4)-*β*-D-digitoxopyransyl-(1→4)-*β*-D-canaropyranoside, cynatratoside B, and glaucogenin C mono-D-thevetoside which were used to identify the monosaccharides obtained in the acid hydrolysis, including their absolute configuration, were isolates from the title plant in our previous work [5]. Acetonitrile used in preparative HPLC procedure was in HPLC grade, and other solvents were of analytical grade.

### 2.2. Plant Material

The roots of *C. stauntonii* were collected from Tongbai County, Henan Province of central China, in August 2011. It was identified by Associate Professor Lin Ma (a savant in plant systematics from Institute of Materia Medica, Chinese Academy of Medical Sciences and Peking Union Medical College). A voucher specimen (ID-S-2426) was deposited in the Herbarium of Institute of Materia Medica, Chinese Academy of Medical Sciences, Beijing, China.

### 2.3. Extraction and Isolation

The dried-up and pulverized roots (30 Kg) of *C. stauntonii* were extracted three times under reflux conditions with 95% EtOH. The combined ethanolic solution was concentrated in vacuo to yield a dark-brown residue (ca. 5000 g). The residue was suspended in 80% aqueous ethanol (ca. 10000 mL) and then extracted with petroleum ether and EtOAc successively in separatory funnel, each for several times until the upper solvent being very transparent. The combined EtOAc solution was washed three times with 5% aqueous solution of NaHCO_3_ (3 × 1000 mL) and then H_2_O (2 × 1000 mL), respectively, to pH 7. After the removal of the organic solvent, 190 g of brown residue was obtained. This resulting residue was fractionated by CC over silica gel eluted with gradient solvents of CHCl_3_–MeOH (100 : 0-1 : 1) to yield 13 fractions (designated as fractions 1 to 13) according to their TLC profiles. Fraction 3 (68 g) was further separated by CC over silica gel using a stepwise gradient solvents of petroleum ether/EtOAc (25 : 1→1 : 1) as eluents to yield seven further subfractions (F3-1-F3-7, also according to the detection of TLC). Fraction F3-5 (7.0 g) was applied to Flash C18 column chromatography eluted with CH_3_OH/H_2_O (40%→100%) to give six subfractions (F3-5-1-F3-5-6). Fraction F3-5-4 (1.1 g) was applied to preparative HPLC system (mobile phase: CH_3_OH/H_2_O (70 : 30, v/v); flow rate: 5 mL min^−1^; UV detection at 210 nm) resulting in the isolation of compound **1 **(36 mg). Fraction F3-6 (3.5 g) was applied to Flash C18 column chromatography eluted with CH_3_OH/H_2_O (40%→100%) to give six subfractions (F3-6-1-F3-6-6). Compound **2** (60 mg) was obtained by recrystallization from F3-6-4. Fraction F3-7 (3.0 g) was applied to Flash C18 column chromatography eluted with CH_3_OH/H_2_O (40%→100%) to give seven subfractions (F3-7-1-F3-7-7). Fraction F3-7-2 (0.2 g) was applied to preparative HPLC system (mobile phase: CH_3_CN/H_2_O (35 : 65, v/v); flow rate: 5 mL min^−1^; UV detection at 210 nm and 280 nm) resulting in the isolation of compound **3 **(12 mg) and compound **4** (22 mg).

The known compound anhydrohirundigenin monothevetoside (**4**) [1] was identified by comparison of their spectroscopic data (^1^H and ^13^C NMR, MS) with the literature values.

#### 2.3.1. Stauntoside L (**1**)

White amorphous powder (CH_3_OH–CHCl_3_), [*α*]_D_^20^ + 17.7 (*c* = 1.14, CH_3_OH, 20°C). IR(KBr) *ν*_max⁡_: 3479, 2933, 1735, 1652, 1452, 1381, 1309, 1162, 1072, 1003, 871, and 606 cm^−1^. ESI-MS (positive mode) *m*/*z*: 817.5 [M+Na]^+^. HRESI-MS (positive mode) *m*/*z*: 817.4002 [M+Na]^+^, calcd for C_41_H_62_O_15_Na, 817.3981. ^1^H NMR (500 MHz, C_5_D_5_N) for aglycone: *δ* 0.77 (3H, s, H-19), 1.53 (3H, s, H-21), 3.54 (1H, d, *J* = 9.0 Hz, H-17), 3.80 (1H, m, H-3), 3.93 (1H, m, H_*β*_-15), 4.23 (1H, t, *J* = 7.7 Hz, H_*α*_-15), 5.32 (1H, d, *J* = 5.0 Hz, H-6), 5.43 (1H, m, H-16), 6.47 (1H, s, H-18). ^1^H NMR (600 MHz, C_5_D_5_N) data of the sugar moiety: see Table 1. ^13^C NMR (150 MHz, C_5_D_5_N): see Table 2.

#### 2.3.2. Stauntoside M (**2**)

White amorphous powder (CH_3_OH–CHCl_3_), [*α*]_D_^20^ + 0.90 (*c* = 1.00, CH_3_OH, 20°C). IR(KBr) *ν*_max⁡_: 3482, 2933, 1733, 1652, 1452, 1382, 1308, 1164, 1077, 1006, 872, and 610 cm^−1^. ESI-MS (positive mode) *m*/*z*: 961.6 [M+Na]^+^. HRESI-MS (positive mode) *m*/*z*: 961.4767 [M+Na]^+^, calcd for C_48_H_74_O_18_Na, 961.4776. ^1^H NMR (500 MHz, C_5_D_5_N) for aglycone: *δ* 0.77 (3H, s, H-19), 1.53 (3H, s, H-21), 3.54 (1H, d, *J* = 8.0 Hz, H-17), 3.82 (1H, m, H-3), 3.94 (1H, m, H_*β*_-15), 4.23 (1H, m, H_*α*_-15), 5.32 (1H, d, *J* = 5.0 Hz, H-6), 5.43 (1H, dd, *J* = 8.0, 17.0 Hz, H-16), 6.47 (1H, s, H-18). ^1^H NMR (500 MHz, C_5_D_5_N) data of the sugar moiety: see Table 1. ^13^C NMR (125 MHz, C_5_D_5_N): see Table 2.

#### 2.3.3. Stauntoside N (**3**)

 White amorphous powder (CH_3_OH-CHCl_3_), [*α*]_D_^20^ + 200.7 (*c* = 1.01, CH_3_OH, 20°C). IR(KBr) *ν*_max⁡_: 3487, 2937, 1682, 1452, 1381, 1326, 1256, 1187, 1061, 1030, 867, 833, 686, and 492 cm^−1^. ESI-MS (positive mode) *m*/*z*: 525.2 [M+Na]^+^. HRESI-MS (positive mode) *m*/*z*: 525.2465 [M+Na]^+^, calcd for C_28_H_38_O_8_Na, 525.2459. ^1^H NMR (600 MHz, C_5_D_5_N) for aglycone: *δ* 0.80 (3H, s, H-19), 1.57 (3H, s, H-21), 2.22 (1H, dd, *J* = 11.4, 5.7 Hz, H-9), 2.78 (1H, d, *J* = 8.4 Hz, H-17), 3.82 (1H, dd, *J* = 10.9, 4.5 Hz, H_*β*_-15), 4.03 (1H, d, *J* = 8.4 Hz, H-18_a_), 4.07 (1H, d, *J* = 8.4 Hz, H-18_b_), 4.28 (1H, br d, *J* = 10.9 Hz, H_*α*_-15), 4.61 (1H, m, H-3), 4.81 (1H, m, H-16), 5.81 (1H, br s, H-4), 5.90 (1H, d, *J* = 9.6 Hz, H-6), 6.64 (1H, d, *J* = 9.6 Hz, H-7). ^1^H NMR (500 MHz, C_5_D_5_N) data of the sugar moiety: see Table 1. ^13^C NMR (125 MHz, C_5_D_5_N): see Table 2.

### 2.4. Acid Hydrolysis of Reference Compounds and Compounds ** 1–3**

Each solution of 6 mg of reference compounds, glaucogenin C 3-*O*-*α*-L-cymaropyranosyl-(1→4)-*β*-D-digitoxopyranosyl-(1→4)-*β*-D-canaropyranoside, cynatratoside B, and glaucogenin C mono-D-thevetoside, and the new compounds **1–3**, was refluxed within 10% HCl (3 mL) at 75°C for 2.5 h. After cooling, the reaction mixture was extracted thoroughly with CHCl_3_, the CHCl_3_ layer was washed with water, and then the water fraction was combined with the original aqueous layer. The aqueous layer was evaporated under vacuum, then rediluted with water and reevaporated in vacuo repeatedly to eliminate the surplus HCl and furnish a final neutral residue. The residue was analyzed by TLC with silica gel G as adsorbents, 10% H_3_PO_4_·12MoO_3_ (phosphomolybdic acid hydrate) in 95% EtOH as detection reagent for spraying, followed by heating the plate to develop the colors, and solvent A, CHCl_3_–CH_3_OH (8-1), and solvent B, EtOAc-acetone (2.5-2) as solvent systems, respectively, for development of sugars. The Rf values of D-digitoxose, D-thevetose, and L-cymarose were determined, by interactive comparison among the three reference compounds, in the order of 0.84, 0.70, and 0.34 over solvent A, and of 0.88, 0.75, and 0.40 over solvent B, respectively. 

### 2.5. Determination of the Absolute Configurations of Monosaccharides

The absolute configurations of D-digitoxose, L-cymarose, and D-thevetose were determined as per the method published by Hara et al. [6]. The monosaccharides obtained on acid hydrolysis, as described above, were dissolved in pyridine and reacted with L-cysteine methyl ester hydrochloride at 60°C for 1 h. Equal volume of acetic anhydride was added and heating was carried out for another 1 h. Acetylated thiazolidine derivatives were injected into GC system. The absolute configurations of the sugars were determined by comparing the retention times with those of acetylated thiazolidine derivatives synthesized from the known sugars obtained through acid hydrolysis of the reference compounds. (Also, the retention times of D-digitoxose, L-cymarose, and D-thevetose were determined by interactive comparison. GC conditions in the test: column, HP-5, 30 m × 0.25 mm, 0.25 *μ*m; detection FID; carrier gas, N_2_; injection temperature, 250°C, detection temperature, 280°C, column temperature, 150°C (0 min), 10°C/min to 250°C (20 min). tR D-digitoxose 13.09 min, tR L-cymarose 13.46 min, and tR D-thevetose 16.07 min).

The D-cymarose involved in this paper was not detected by GC method because of the lack of reference sugars, but, from the results of the typical monosaccharides, it can be concluded that the absolute configurations of the monosaccharides composed of the sugar units can be really determined by comparison of their spectroscopic data with those reported in the literature. This determination is also because of the very common kind of D-cymarose in the case of the *Cynanchum* species.

## 3. Results and Discussion

All three new compounds were obtained as white lamellae or amorphous powder and showed up positive Liebermann-Burchard and Keller-Kiliani reactions, suggesting their glycosidic steroidal category with 2-deoxysugar units existing in their sugar moieties [7].

### 3.1. Stauntoside L (**1** )

**Figure 2:**
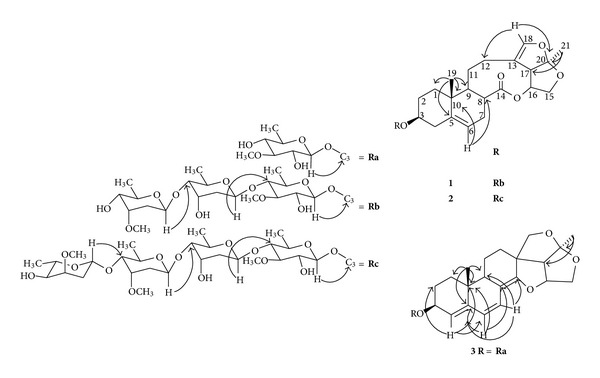
Principal HMBC correlations of the new compounds (**1–3**). (→: HMBC).

The positive HRESI-MS of **1** gave a pseudomolecular ion peak at *m/z* 817.4002 [M+Na]^+^, corresponding to the molecular formula C_41_H_62_O_15_. The IR spectrum showed the absorption bands for hydroxy (3479 cm^−1^), carbonylic (1735 cm^−1^), and olefinic (1652 cm^−1^) groups. The ^1^H NMR spectrum of **1** revealed the diagnostic signals of steroidal glycoside, with a 13,14 : 14,15-disecopregnane-type skeleton aglycone being exhibited by two tertiary methyls resonated at *δ* 0.77 (3H, s, H-19) and 1.53 (3H, s, H-21) and one methyleneoxy group resonated at *δ* 3.93 (1H, m, H_*β*_-15) and 4.23 (1H, t, *J* = 7.7 Hz, H_*α*_-15), and with three sugar units being shown by three anomeric proton signals at *δ* 4.81 (1H, d, *J* = 7.5 Hz, H-1′), 5.51 (1H, dd, *J* = 9.5, 1.5 Hz, H-1′′), and 5.11 (1H, dd, *J* = 10.0, 1.5 Hz, H-1′′′), which correlated to the corresponding anomeric carbon signals at *δ*_C_ 102.3 (C-1′), 99.0 (C-1′′), and 99.8 (C-1′′′), respectively, in the HSQC spectrum, and three secondary methyls at *δ* 1.45 (3H, d, *J* = 6.5 Hz, H-6′), 1.42 (3H, d, *J* = 6.5 Hz, H-6′′), and 1.46 (3H, d, *J* = 6.0 Hz, H-6′′). In addition, two characteristic olefinic proton signals at *δ* 5.32 (1H, d, *J* = 5.0 Hz, H-6) and 6.47 (1H, s, H-18) and two methoxyls at *δ* 3.44 (3H, s) and 3.94 (3H, s) were also determined in the ^1^H NMR spectrum, the later olefinic signal was obviously deshielded and the two methoxyls were compatible with two methylated deoxypyranoses when examining the ^13^C and DEPT NMR data which exhibited forty-one carbon signals, with seven methyls, nine methylenes, twenty methines, and five quaternary carbons being categorized (Table 2). With the exception of the ^13^C and DEPT NMR signals assignable to three monosaccharides, the remaining resonances were very similar to those of glaucogenin C, a known steroidal aglycone isolated previously from *C. atratum *[8]. The main differences were observed for glycosidation shifts at C-2 (−2.3), C-3 (+7.1), and C-4 (−4.0) in aglycone moiety of **1**, so the oligosaccharide chain was determined to link with the C-3 hydroxyl of **1**, which was also confirmed, with the aid of HSQC spectrum for determining the direct carbon-proton linkages, by the long-range ^1^H–^13^C correlation of the signal of H-1′ with the signal of C-3 in the HMBC spectrum. After the anomeric protons were identified, the ^1^H–^1^H COSY experiment, coupled with the HSQC spectrum, was very effective in determining the spin systems within the sugar moieties because of the handsome differences of the chemical shifts and the relatively large coupling constants theoretically (Figure 1). One *β*-D-thevetopyranose, one *β*-D-digitoxopyranose, and one *β*-D-cymaropyranose in the very three sugar units were further speculated by comparing the ^1^H and ^13^C NMR spectroscopic data of **1** with those of stauntoside J [5], which were supported by the splitting patterns and coupling constants of the above-mentioned anomeric proton signals. These conclusions about the absolute configurations of the monosaccharides were confirmed by acid hydrolysis as described above in Acid Hydrolysis of Reference Compounds and Compounds **1–3** and Determination of the Absolute Configurations of Monosaccharides, which not only gave one D-thevetopyranose, one D-digitoxopyranose, and another kind of sugar unit, but also confirmed that the absolute configurations of the monosaccharides determined by comparison of their spectroscopic data with those reported are really consistent with reality. Also, this determination is because of the very common kind of D-cymarose in the case of the *Cynanchum* species. Because of the lack of reference substance, D-cymaropyranose units could not be determined in the GC test. The sugar sequence of **1** was demonstrated by HMBC correlations from *δ*_H_ 5.11 (H-1′′′ of *β*-D-cymaropyranose) to *δ*_C_ 83.2 (C-4′′ of *β*-D-digitoxopyranose), from *δ*_H_ 5.51 (H-1′′ of *β*-D-digitoxopyranose) to *δ*_C_ 82.9 (C-4′ of *β*-D-thevetopyranose), and from *δ*_H_ 4.81 (H-1′ of *β*-D-thevetopyranose) to *δ*_C_ 78.2 (C-3) (Figure 2). Thus, compound **1** was established to be glaucogenin C 3-*O*-*β*-D-cymaropyranosyl-(1→4)-*β*-D-digitoxopyranosyl-(1→4)-*β*-D-thevetopyranoside and was given the trivial name of stauntoside L.

### 3.2. Stauntoside M (**2**)

The positive HRESI-MS of**2** gave a pseudomolecular ion peak at *m/z* 961.4767 [M+Na]^+^, corresponding to the molecular formula C_48_H_74_O_18_. The IR spectrum showed the absorption bands for hydroxy (3482 cm^−1^), carbonylic (1733 cm^−1^), and olefinic (1652 cm^−1^) groups. A detailed comparison between compounds **2 **and** 1** indicated that they have the consistent ^1^H- and ^13^C-NMR spectroscopic data from their aglycone moieties (see experimental and Table 2), which was confirmed to be glaucogenin C by detailed analysis of 2D NMR spectra (Figure 2. Complete data not shown). With the exception of the aglycone signals, the ^1^H NMR spectrum of **2** revealed the diagnostic signals of four sugar units by four anomeric proton signals at *δ* 4.82 (1H, d, *J* = 8.0 Hz, H-1′), 5.52 (1H, br d, *J* = 10.0 Hz, H-1′′), 5.13 (1H, br d, *J* = 9.5 Hz, H-1′′′), and 5.19 (1H, d, *J* = 3.5 Hz, H-1′′′′), which correlated to the corresponding anomeric carbon signals at *δ*_C_ 102.3 (C-1′), 99.0 (C-1′′), 99.6 (C-1′′′), and 101.2 (C-1′′′′), respectively, in the HSQC spectrum, and four secondary methylic signals at *δ* 1.46 (3H, d, *J* = 6.0 Hz, H-6′), 1.42 (3H, d, *J* = 6.0 Hz, H-6′′ or H-6′′′′), 1.30 (3H, d, *J* = 6.5 Hz, H-6′′′), and 1.56 (3H, d, *J* = 6.5 Hz, H-6′′′′ or H-6′′). The ^1^H, ^1^H-COSY experiment, coupled with the HSQC spectrum, established the spin systems within the sugar moiety (Figure 1). By comparing the ^1^H- and ^13^C-NMR spectroscopic data of **2** with those of **1** and stauntoside H [5], the structures of the four sugar units were suggested, that is, one *β*-D-thevetopyranose, one *β*-D-digitoxopyranose, one *β*-D-cymaropyranose, and one *α*-L-cymaropyranose, which were further supported by the splitting patterns of the above-mentioned anomeric proton signals. Compound **2** was subjected to acid hydrolysis and GC analysis as described above in Acid Hydrolysis of Reference Compounds and Compounds **1–3** and Determination of the Absolute Configurations of Monosaccharides, which gave D-thevetopyranose, D-digitoxopyranose and L-cymaropyranose, and another kind of sugar unit. Because of the lack of reference substance, D-cymaropyranose unit could not be determined in the GC test. The linkages of the four sugars were ascertained by the HMBC spectrum, which showed long-range ^1^H–^13^C correlations from *δ*_H_ 5.19 (H-1′′′′ of *α*-L-cymaropyranose) to *δ*_C_ 82.3 (C-4′′′ of *β*-D-cymaropyranose), from *δ*_H_ 5.13 (H-1′′′ of *β*-D-cymaropyranose) to *δ*_C_ 83.2 (C-4′′ of *β*-D-digitoxopyranose), from *δ*_H_ 5.52 (H-1′′ of *β*-D-digitoxopyranose) to *δ*_C_ 82.9 (C-4′ of *β*-D-thevetopyranose), and from *δ*_H_ 4.82 (H-1′ of *β*-D-thevetopyranose) to *δ*_C_ 78.2 (C-3) (Figure 2). Hence, the structure of compound **2** was elucidated to be glaucogenin C 3-*O*-*α*-L-cymaropyranosoyl-(1→4)-*β*-D-cymaropyranosoyl-(1→4)-*β*-D-digitoxopyranosoyl-(1→4)-*β*-D-thevetopyranoside and was given the trivial name of stauntoside M.

### 3.3. Stauntoside N (**3**)

Compound **3** was determined to possess the molecular formula C_28_H_38_O_8_ by its pseudomolecular ion peak at *m*/*z* 525.2465 [M + Na]^+^ in the positive HRESI-MS experiment. The IR spectrum showed the absorption bands for hydroxy (3487 cm^−1^) and olefinic (1682 cm^−1^) groups. The ^1^H NMR spectroscopic data of **3** (see experimental and Table 1) revealed the diagnostic signals of steroidal glycoside, with an aglycone of 14,15-secopregnane-type skeleton being exhibited by two tertiary methyls resonated at *δ* 0.80 (3H, s, H-19) and 1.57 (3H, s, H-21), two methineoxy groups at *δ* 4.61 (1H, m, H-3) and 4.81 (1H, m, H-16), and two methyleneoxy groups at *δ* 3.82 (1H, dd, *J* = 10.9, 4.5 Hz, H_*β*_-15) and 4.28 (1H, br d, *J* = 10.9 Hz, H_*α*_-15), and at *δ* 4.03 (1H, d, *J* = 8.4 Hz, H-18_a_) and 4.07 (1H, d, *J* = 8.4 Hz, H-18_b_), and with one sugar unit being shown by one anomeric proton signal at *δ* 4.90 (1H, d, *J* = 7.8 Hz, H-1′), which correlated to the anomeric carbon signal at *δ*_C_ 103.5 (C-1′) in the HSQC spectrum, and one secondary methyl at *δ* 1.62 (3H, d, *J* = 6.0 Hz, H-6′). In addition, three characteristic olefinic proton signals at *δ* 6.64 (1H, d, *J* = 9.6 Hz, H-7), 5.90 (1H, d, *J* = 9.6 Hz, H-6), and 5.81 (1H, s, H-4) and one methoxyl at *δ* 3.92 (3H, s) were also determined. The ^13^C and DEPT NMR spectra exhibited twenty-eight carbon signals, with four methyls, six methylenes, twelve methines, and six quaternary carbons being categorized (Table 2). Comparison of ^1^H and ^13^C NMR spectroscopic data of **3** with those of stauntoside C [5], as well as the information obtained from HSQC experiments, demonstrated that most signals of **3** were superimposable to its counterparts in stauntoside C, except for the sugar unit. On acid hydrolysis, **3** afforded thevetose. The absolute configuration of thevetose was determined to be D-type through GC analysis as described above in *2.4* and *2.5*. Coupled with the coupling constant of the anomeric proton, the sugar unit was solidly determined to be *β*-D-thevetopyranose. Furthermore, by comparing with anhydrohirundigenin monothevetoside [1] and glaucogenin-C *β*-D-thevetopyranoside [9], the HMBC experiment confirmed the connectivities in compound **3 **which showed the significant long-range ^1^H–^13^C correlations from *δ*_H_ 4.90 (H-1′) to *δ*_C_ 75.4 (C-3), and from *δ*_H_ 4.61 (H-3) to *δ*_C_ 103.5(C-1′) (Figure 2). Therefore, compound **3** was elucidated as deoxyamplexicogenin A 3-*O*-*β*-D-thevetopyranoside and was given the trivial name of stauntoside N.

## 4. Conclusions

In recent years, only several papers have described phytochemical investigations of *C. stauntonii* and led to a small amount of steroidal glycosides being reported. In the present work, we reported on three new steroidal glycosides, named as stauntosides L, M, and N, from *C. stauntonii*. Here, the structure elucidation, mainly undertaken by means of spectroscopic and chemical evidence, provided unambiguous information about the aglycone skeletons and structures, the position of the glycosidic linkage, and the sequence of the monosaccharides in the sugar moiety. In addition, it should be emphasized that the main and active ingredients of *Cynanchum *species are steroidal glycosides [10]. In conclusion, this study has enriched the information about the compounds of the title plant and further established that *C. stauntonii* is a significant source of steroidal glycosides.

## Figures and Tables

**Table 1:** The ^1^H NMR chemical shifts of the sugar moieties of compounds **1**–**3 **in C_5_D_5_N (**δ** in ppm, *J* values in Hz).

H	**1** ^ a^	**2** ^ a^	**3** ^ b^
	*β*-D-the	*β*-D-the	*β*-D-the
1′	4.81 d(7.5)	4.82 d(8.0)	4.90 d(7.8)
2′	3.93	3.94	3.99
3′	3.68	3.71	3.67
4′	3.72	3.70	3.67
5′	3.66	4.21	3.77
6′	1.45 d(6.5)	1.46 d(6.0)	1.62 d(6.0)
3′-OCH_3_	3.94 s	3.94 s	3.92 s

	*β*-D-digt	*β*-D-digt	
1′′	5.51 dd(9.5,1.5)	5.52 d(10.0)	
2′′	1.68, 2.35	2.01, 2.43	
3′′	3.71	4.64	
4′′	3.49	3.48 dd(9.5,2.5)	
5′′	4.30	4.31	
6′′	1.42 d(6.5)	1.42 d(6.0)*	
3′′-OCH_3_			

	*β*-D-cym	*β*-D-cym	
1′′′	5.11 dd(10.0,1.5)	5.13 d(9.5)	
2′′′	2.00, 2.42	1.67, 2.40	
3′′′	4.64	3.92	
4′′′	3.47	3.39 dd(9.5,2.5)	
5′′′	4.11	3.67	
6′′′	1.46 d(6.0)	1.30 d(6.5)	
3′′′-OCH_3_	3.44 s	3.52 s	

		*α*-L-cym	
1′′′′		5.19 d(3.5)	
2′′′′		2.07, 2.38	
3′′′′		3.85	
4′′′′		4.06	
5′′′′		4.31	
6′′′′		1.56 d(6.5)*	
3′′′′-OCH_3_		3.31 s	

*Not differentiated.

^a^500 MHz; ^b^600 MHz.

the: thevetopyranosyl; digit: digitoxopyranosyl; cym: cymaropyranosyl.

**Table:** (a)

C	Aglycon moiety		
	**1** ^ a^	**2** ^ a^	**3** ^ b^
1	36.5 t	36.5 t	33.6 t
2	30.0 t	30.0 t	27.8 t
3	78.2 d	78.2 d	75.4 d
4	39.1 t	39.0 t	125.2 d
5	140.6 s	140.6 s	144.5 s
6	120.4 d	120.4 d	125.7 d
7	30.0 t	30.0 t	122.6 d
8	40.7 d	40.7 d	108.2 s
9	53.2 d	53.2 d	44.2 d
10	38.7 s	38.7 s	35.6 s
11	23.9 t	23.9 t	20.5 t
12	28.4 t	28.4 t	30.8 t
13	118.5 s	118.5 s	54.9 s
14	175.5 s	175.5 s	155.3 s
15	67.8 t	67.8 t	72.1 t
16	75.5 d	75.5 d	86.2 d
17	56.2 d	56.2 d	62.1 d
18	143.8 d	143.8 d	77.5 t
19	17.8 q	17.8 q	17.7 q
20	114.4 s	114.4 s	118.5 s
21	24.8 q	24.8 q	22.7 q

**Table:** (b)

C	Sugar moiety		
	**1** ^ a^	**2** ^ a^	**3** ^ b^
	*β*-D-the	*β*-D-the	*β*-D-the
1′	102.3 d	102.3 d	103.5 d
2′	74.6 d	74.6 d	75.1 d
3′	85.8 d	85.8 d	88.2 d
4′	82.9 d	82.9 d	76.0 d
5′	71.6 d	71.6 d	72.8 d
6′	18.7 q	18.7 q	18.7 q
3′-OCH_3_	60.5 q	60.5 q	61.0 q

	*β*-D-digt	*β*-D-digt	
1′′	99.0 d	99.0 d	
2′′	39.0 t	39.1 t	
3′′	67.7 d	67.7 d	
4′′	83.2 d	83.2 d	
5′′	68.8 d	68.8 d	
6′′	18.5 q	18.5 q*	

	*β*-D-cym	*β*-D-cym	
1′′′	99.8 d	99.6 d	
2′′′	35.6 t	34.9 t	
3′′′	78.8 d	77.4 d	
4′′′	74.1 d	82.3 d	
5′′′	71.0 d	69.3 d	
6′′′	18.9 q	18.6 q	
3′′′-OCH_3_	58.0 q	57.3 q	

		*α*-L-cym	
1′′′′		101.2 d	
2′′′′		30.9 t	
3′′′′		75.8 d	
4′′′′		67.5 d	
5′′′′		67.7 d	
6′′′′		17.8 q*	
3′′′′-OCH_3_		55.0 q	

^∗^not differentiated.

^a^125 MHz; ^b^150 MHz.

the: thevetopyranosyl; digit: digitoxopyranosyl; cym: cymaropyranosyl.
